# The future of cancer therapy: exploring the potential of patient-derived organoids in drug development

**DOI:** 10.3389/fcell.2024.1401504

**Published:** 2024-05-20

**Authors:** Cigir Biray Avci, Bakiye Goker Bagca, Behrouz Shademan, Leila Sabour Takanlou, Maryam Sabour Takanlou, Alireza Nourazarian

**Affiliations:** ^1^ Department of Medical Biology, Faculty of Medicine, Ege University, Izmir, Türkiye; ^2^ Department of Medical Biology, Faculty of Medicine, Adnan Menderes University, Aydın, Türkiye; ^3^ Stem Cell Research Center, Tabriz University of Medical Sciences, Tabriz, Iran; ^4^ Department of Basic Medical Sciences, Khoy University of Medical Sciences, Khoy, Iran

**Keywords:** patient-derived organoids, cancer therapy, drug development, personalized medicine, preclinical drug screening

## Abstract

Cancer therapy is on the brink of a significant transformation with the inclusion of patient-derived organoids (PDOs) in drug development. These three-dimensional cell cultures, directly derived from a patient’s tumor, accurately replicate the complex structure and genetic makeup of the original cancer. This makes them a promising tool for advancing oncology. In this review, we explore the practical applications of PDOs in clinical drug screening and pharmacognostic assessment, as well as their role in refining therapeutic strategies. We provide insights into the latest advancements in PDO technology and its implications for predicting treatment responses and facilitating novel drug discoveries. Additionally, we address the operational challenges associated with incorporating PDOs into the drug development process, such as scaling up organoid cultures, ensuring consistent results, and addressing the ethical use of patient-derived materials. Aimed at researchers, clinicians, and key stakeholders in oncology, this article aims to succinctly present both the extraordinary potential and the obstacles to integrating PDOs, thereby shedding light on their prospective impact on the future of cancer treatment.

## 1 Introduction

Cancer remains a significant health challenge in the 21st century, with its worldwide impact continuing to grow. Due to the complexity and genetic diversity of the disease, there is an increasing trend in the scientific community toward personalized treatment strategies. Patient-derived organoids (PDOs) have emerged as a groundbreaking tool in oncology research (Sachs and Clevers, 2014), offering three-dimensional cell cultures that better resemble human cancer compared to traditional two-dimensional methods (Weeber et al., 2017). These mini-organs preserve the histological and genetic characteristics of the original tumors (van de Wetering et al., 2015), making them invaluable for studying tumor biology, progression, and treatment response in a controlled laboratory setting. This helps bridge the gap between preclinical models and clinical outcomes (Vlachogiannis et al., 2018).

Historically, drug development has faced challenges such as high costs and low success rates, with many compounds failing in clinical trials due to inefficacy or unexpected toxicity (DiMasi et al., 2016). PDOs represent a paradigm shift by providing a more predictive model for evaluating the drug efficacy and toxicity. This patient-centered approach also enables the exploration of interpatient variability, which is crucial for personalized medicine (Wensink et al., 2021). The application of PDOs in drug discovery is multifaceted, involving high-throughput screenings to assess compound libraries against a diverse range of organoids (Drost and Clevers, 2018).

**FIGURE 1:**
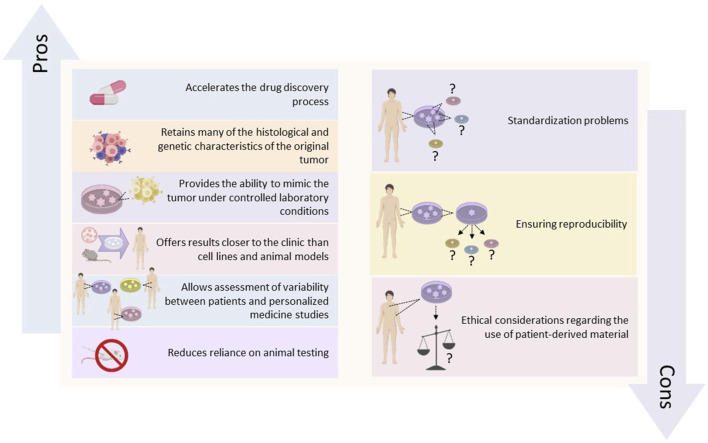
Advantages and disadvantages of PDOs in cancer research.

This strategy is complemented by co-clinical trials, where organoids derived from a patient’s tumor are used alongside clinical treatment to administer the most effective therapy (Servant et al., 2021). Additionally, it includes the potential for co-clinical trials and where organoids dеrivеd from a patiеnt’s tumor arе used in parallel with the patient’s clinical treatment to identify the most еffеctivе thеrapy for that individual (Fujii et al., 2016). These approaches not only expedite the drug discovery process but also improve the ethical aspects of cancer research by minimizing animal testing and focusing on human models (Tuveson and Clevers, 2019). Moreover, PDOs contribute significantly to understanding drug resistance mechanisms, as researchers can observe resistance patterns and explore combination therapies (Shaffer et al., 2017). They also facilitate the study of the tumor microenvironment, which greatly influences drug sensitivity and resistance (Katt et al., 2016). The advantages and disadvantages of PDOs in cancer research are shown in Figure 1. Despite these advantages, the widespread implementation of PDOs in drug development faces practical challenges such as standardizing organoid culture to ensure reproducibility and scalability (Boj et al., 2015). Ethical considerations related to the use of patient-derived materials, such as consent and privacy, also require attention (Munsie et al., 2017).

To use the full potential of PDOs in cancer therapy, a multidisciplinary approach is essential, necessitating collaboration across various fields, including oncology, biology, pharmacology, and ethics. This article provides an overview of the current state of PDOs in drug development, acknowledging both the obstacles and the promising directions that could revolutionize cancer treatment.

## 2 Historical perspective

Cancer is an unyielding and intricate adversary. It has been extensively examined using various research models, including traditional *in vitro* cell lines (Sajjad et al., 2021) and *in vivo* animal models (Abdolahi et al., 2022) These models have played a crucial role in our initial comprehension of cancer biology and therapeutics. While cell lines offer valuable insights into cancer cell behavior, they often fail to replicate the three-dimensional structure and tumor microenvironment observed *in vivo* (Barbosa et al., 2021). Animal models, particularly murine ones, provide a systemic perspective but encounter challenges in translating findings to clinical settings due to interspecies differences (Onaciu et al., 2020; Shin et al., 2021).

The emergence of organoids has revolutionized cancer research by providing three-dimensional cultures derived from stem cells or patient tumors. These cultures closely mimic the architecture and function of organs, offering a relevant platform for studying cancer biology and therapy (Clevers and Tuveson, 2019). The use of cancer cells cultivated in three-dimensional environments has yielded clinically pertinent data, reflecting the tumor environment of patients (Fessart and Delom, 2024). Personalized medicine has especially benefited from the development of PDOs, which accurately replicate the genetic, transcriptomic, and histopathological characteristics of the patient’s tumor. PDOs serve as effective tools for drug screening and predicting therapeutic responses (Xu et al., 2019a). Advancements such as integration of CRISPR–Cas9 technology with organoid cultures have further propelled the field by enabling precise genetic manipulation and facilitating the study of gene function and drug–gene interactions within a relevant tumor context (Hendriks et al., 2021). High-throughput screening technologies have expedited the identification of potential therapeutic agents (Tuveson and Clevers, 2019). Furthermore, the inclusion of immune cells in co-culture systems has provided invaluable insights into the tumor immune microenvironment, which is essential for development of immunotherapies (Campillo et al., 2019). The establishment of biobanks for organoid lines, ensuring a diverse representation of tumor subtypes and patient demographics, has been another significant stride, providing valuable resources for ongoing and future oncological studies (Tuveson and Clevers, 2019).

In conclusion, the integration of organoid technology into oncology research marks a significant shift in drug development. This approach sets the stage for a new era of personalized cancer therapy, with the potential to bridge the gap between preclinical studies and clinical application, thereby enhancing outcomes for cancer patients worldwide.

## 3 Biotechnological advances in organoid culturing

PDOs represent a significant breakthrough in cancer research, utilizing the inherent ability of stem cells to self-organize into three-dimensional (3D) structures. These structures emulate the intricate architecture and function of native tissues (Kretzschmar, 2021). The process involves harvesting stem cells from a patient’s tumor biopsy, which are then cultured in specialized scaffolds that support 3D growth, along with growth factors that simulate the natural cellular environment. The resulting organoids retain the genetic and phenotypic characteristics of the original tumor (Drost and Clevers, 2018), marking an important milestone in personalized medicine. Advancements in the field have been propelled by improved culturing methods, such as the use of the Matrigel dome technique and the development of synthetic and tunable hydrogels, including alginate- and collagen-based matrices (Tuveson and Clevers, 2019; Fang et al., 2023). These innovations offer controlled environments that enhance reproducibility across different research settings (Tuveson and Clevers, 2019). Additionally, the integration of microfluidic systems with precise microchannels has streamlined the automated delivery of nutrients and waste management, optimized growth conditions, and enabled large-scale, high-throughput drug screening (El Harane et al., 2023). 3D bioprinting technology has further advanced organoid complexity by allowing precise placement of cells and matrix components in a layer-by-layer fashion (Drost and Clevers, 2018; Tuveson and Clevers, 2019; Fang et al., 2023). This creates intricate and diverse structures that more accurately reflect the tumor microenvironment, including vascular networks, which are crucial for studying the effects of anticancer drugs targeting the tumor vasculature (Zhao et al., 2021).

The co-culture capability of bioprinting permits various cell types, such as cancer, stromal, and immune cells, to be cultured together, generating a more precise model of tumor–immune system interactions—a key aspect in immunotherapy research (Drost and Clevers, 2018; Tiwari et al., 2022). These advancements are transforming preclinical drug development by providing models that more closely resemble patient tumors, enhancing the accuracy of predictions regarding drug effectiveness and resistance, which could lead to tailored treatment plans (Lim et al., 2023). For instance, researchers have successfully bioprinted glioblastoma organoids from patients, which incorporate endothelial cells, facilitating personalized evaluations of responses to anti-angiogenic therapies (Zhang et al., 2023). This approach not only has the potential to improve treatment outcomes but also reduce the time and costs associated with traditional drug development processes. Despite these advancements, it is crucial to address the challenges that persist in fully harnessing the potential of these technologies. Broad adoption in research and clinical settings requires consideration of factors such as cost, accessibility, and standardization (Tu et al., 2023). Moreover, further research is needed to refine bioprinting techniques for scalability and to ensure the long-term viability and functionality of complex organoids (Hafa et al., 2023). In summary, recent innovations in organoid culturing technologies, including synthetic hydrogels, microfluidic systems, and 3D bioprinting, are revolutionizing preclinical cancer research. These technologies facilitate the development of more complex and patient-specific models that could significantly advance personalized cancer therapy by overcoming limitations of traditional cultures and providing more accurate representations of tumor biology.

## 4 Organoids in cancer research: current applications

**FIGURE 2:**
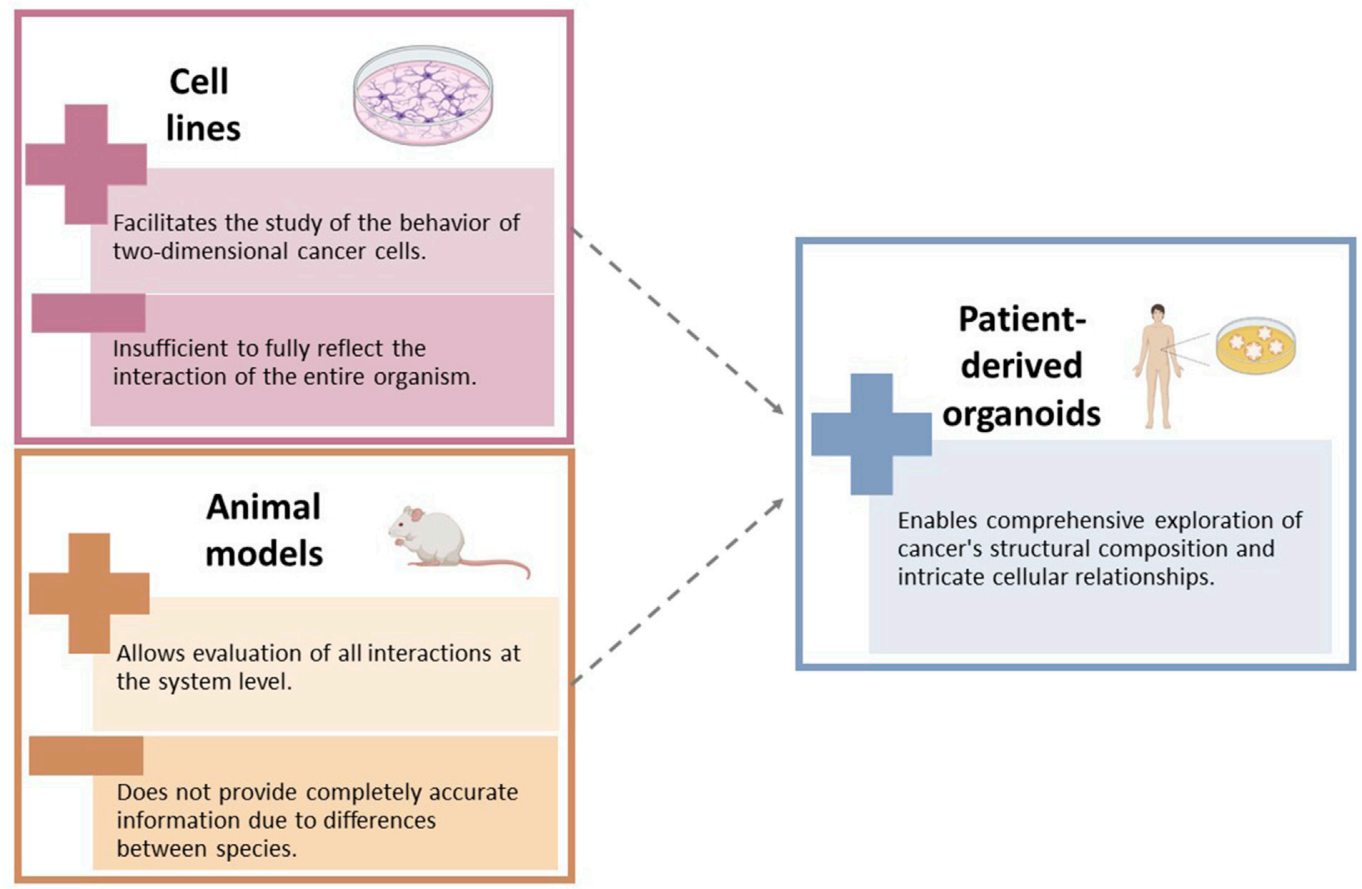
Limitations of the use of cell lines and animal models in cancer research.

PDOs have played a crucial role in cancer research by bridging the gap between basic *in vitro* cell lines and complex *in vivo* animal models. Figure 2 show the limitations of the use of cell lines and animal models in cancer research. These three-dimensional, multicellular systems accurately replicate the structural and functional characteristics of human tumors, making them indispensable for personalized medicine and the study of cancer progression, metastasis, and treatment response (Sachs et al., 2018). PDOs maintain the genetic and phenotypic features of the original tumor, allowing for precise evaluation of individual drug responses and the development of personalized treatments (Drost and Clevers, 2018). They also provide insights into the dynamics of cancer progression and the role of the microenvironment, including the influence of stromal and immune cells (Nagini, 2017).

For instance, pancreatic cancer PDOs have provided valuable information about tumor invasion and metastasis, revealing that elevated levels of doublecortin-like kinase 1 (DCLK1) can drive cancer cell migration. Additionally, targeting DCLK1 may inhibit liver metastasis, suggesting potential new therapeutic targets (Ito et al., 2016). Moreover, these organoids are instrumental in drug screening and preclinical testing, expediting the discovery of novel cancer treatments. Organoids derived from mammary Paget’s disease (MPD) have been particularly beneficial for breast cancer research as they accurately represent the genomic landscape of the disease, including copy number variations, mutational burden, and specific mutations in cancer-related genes identified through extensive genomic sequencing. Consequently, MPD organoids serve as an effective *ex vivo* platform for studying both the clinical and genomic aspects of breast cancer (Yu et al., 2022). In the context of glioblastoma, patient-derived orthotopic xenografts (PDOX) and the resulting organoids retain the key characteristics of the original tumor, making them suitable for drug screening and predicting therapeutic responses. Studies have shown that glioblastoma organoids respond to treatments such as temozolomide and targeted therapies, thus paving the way for personalized interventions based on genetic and epigenetic markers (Xu et al., 2019b; Yu et al., 2022). For example, researchers have used glioblastoma PDOs to identify potential new drugs. These drugs are currently undergoing evaluation in clinical trials (Rahman et al., 2023).

**FIGURE 3:**
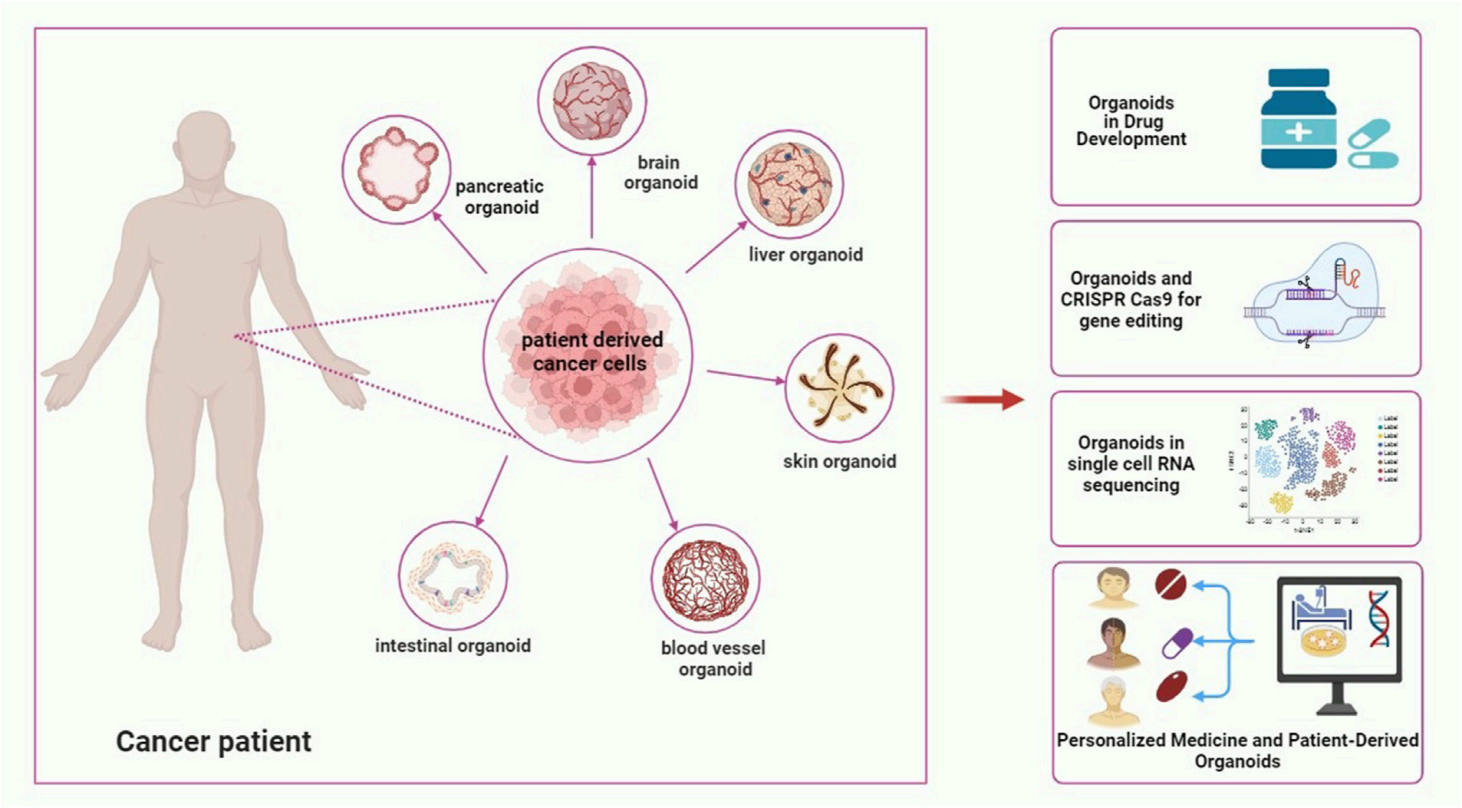
Patient-derived organoids in current and future applications.

Technological advancements in culturing methods and bioengineering are propelling the research field forward. Novel extracellular matrix analogs, microfluidic systems, and 3D bioprinting are improving organoid viability, function, and structure, enabling the creation of more sophisticated models for tumor studies and personalized medicine (Xu et al., 2019b; Yu et al., 2022). Additionally microfluidic systems enable dynamic culturing with continuous nutrient supply and waste removal and further improving the physiological relevance of these models (Babaliari et al., 2023). Figure 3 illustrates the current and future applications of PDOs.

However, challenges such as scalability, cost, and standardization persist. Efforts are underway to incorporate immune cells and capture the full spectrum of tumor heterogeneity in PDOs (Xu et al., 2019b; Ding et al., 2021). In conclusion, PDOs are revolutionizing cancer research by providing models that closely resemble human tumors. The integration of cellular biology and cutting-edge bioengineering holds great promise for the future of personalized therapies and targeted treatments. Continuous technological advancements and addressing current limitations are essential for PDOs to potentially transform cancer treatment and significantly improve patient outcomes.

## 5 Organoids in drug development

PDOs have significantly transformed high-throughput screening (HTS) in cancer therapy by facilitating the swift assessment of multiple anticancer drugs on tumor tissues that closely mimic the *in vivo* environment (Driehuis et al., 2020). These 3D cultures, derived from cancer cells of patients, accurately represent the genetic and phenotypic variations found in tumors. They serve as personalized models for developing drugs and therapies in the field of oncology (Wei et al., 2021). They represent a shift away from a generic approach to targeted treatment strategies, significantly enhancing the precision and effectiveness of HTS. For instance, studies have shown that glioblastoma organoids can accurately predict clinical responses to chemotherapy agents, which could potentially reduce the time and costs involved in drug development (Driehuis et al., 2019).

Furthermore, the integration of organoids with pharmacogenomics is driving the advancement of personalized medicine in the field of oncology. Research has consistently demonstrated the considerable value of PDOs in identifying genetic factors that contribute to drug response and resistance (Rajan et al., 2023). This has led to the development of customized treatment plans aimed to improve treatment effectiveness and minimize side effects.

In 2019, research utilizing PDOs identified specific genomic alterations that correlate with drug sensitivity or resistance, which further underscores the potential of PDOs in tailoring cancer treatment to an individual’s genetic profile (Driehuis et al., 2019). Success stories have documented the pivotal role of organoids in facilitating drug development. An example of this is the utilization of the therapeutically guided multidrug optimization (TGMO) technique in colorectal cancer models. This approach has proven effective in identifying optimized drug combinations that surpass the efficacy of conventional care (Cheng et al., 2022).

In another study, Neal et al. presented an air–liquid interface (ALI) method that enables the growth of PDOs from tumors in both humans and mice. This method preserves the tumor epithelium and the native immune cells (T, B, NK, and macrophages) that are present within the tumor. Consequently, the PDOs accurately replicate the original tumor’s T-cell receptor (TCR) spectrum (Neal et al., 2018). Despite these advancements, challenges such as cost, scalability, and standardization remain in the use of PDOs for drug development. Active research areas include incorporating the complexity of the tumor microenvironment and immune system components. Addressing these hurdles will be essential for wider adoption and full realization of PDOs’ transformative potential in cancer treatment (Genova et al., 2021; Lv et al., 2022).

## 6 Personalized medicine and patient-derived organoids

Personalized medicine in oncology is undergoing a significant transformation with the use of PDOs. These PDOs play a crucial role in developing individualized treatment strategies by cultivating a patient’s own tumor cells, which accurately represent the specific genetic and molecular properties of their cancer. This precision approach not only enhances treatment efficacy but also reduces side effects and the use of ineffective medications (Wang et al., 2022a; Zeng et al., 2023). Evidence strongly supports the use of PDOs for pre-treatment testing, as studies have shown higher response rates and improved survival outcomes compared to conventional treatments that do not consider individual variability (Yahng et al., 2017; Wensink et al., 2021). PDOs serve as reliable *in vitro* models that simulate the patient’s tumor environment, providing a predictive framework for understanding how a tumor may respond to specific therapies (Dantes et al., 2023). Integrating genomic data with organoid responses holds promise in creating predictive models, especially for rare or aggressive cancer types with limited treatment options. For example, a 2021 study used PDOs to forecast responses to immunotherapy in melanoma patients, helping identify those most likely to benefit from such treatments (Abbott et al., 2021).

While PDO technology has vast potential, it is crucial to address the ethical implications inherent in patient-centered medicine. Issues such as ownership of biological material, commercial use of organoids, informed consent, and genetic privacy must be carefully navigated. Clearly defined consent processes and ethical guidelines are essential to maintaining patient trust and effectively managing unexpected genetic findings that could impact patients or their relatives (de Jongh et al., 2022). It is crucial to maintain trust and intеgrity within the patient–physician relationship and the broader medical community. Although PDOs have immense potential, challenges still exist in fully realizing their clinical utility for personalized medicine. Cost and scalability and potential discrepancies between *in vitro* and *in vivo* responses are key limitations that require attention (Goetz and Schork, 2018). Furthermore, despite the potential of PDOs, there are practical hurdles that hinder their widespread clinical application. Challenges such as cost, scalability, and disparities between *in vitro* and *in vivo* responses are significant and are actively being addressed by the scientific community. Ongoing research aims to improve the scalability and standardization of PDOs, and the integration of complementary technologies like microfluidics and artificial intelligence (AI) is paving the way for broader implementation and more sophisticated applications.

In conclusion, PDOs offer a transformative avenue for cancer treatment and drug development. Their unique ability to replicate individual tumor biology and predict treatment response brings us closer to a future of more precise and effective cancer therapies. Addressing ethical considerations and overcoming current obstacles are key to unlocking the full potential of this innovative technology.

## 7 Challenges and limitations

PDOs hold tremendous potential in the fields of personalized medicine and drug development. However, there are several challenges that need to be addressed through further research. One significant challenge is maintaining the inherent heterogeneity and complexity of tumors within PDOs. A single PDO model may not fully capture the complexity of an entire tumor because intra-tumor heterogeneity can lead to varying growth patterns and drug responses, making clinical applications more complicated (Ramon et al., 2020; Bose et al., 2021). Another crucial aspect is long-term viability and functionality, which require a deep understanding of the tumor microenvironment, including stromal interactions and nutrient signaling (Gjorevski et al., 2016; Chitrangi et al., 2023).

Standardization is also a persistent concern, as variations in protocols, starting materials, and culture media compositions can impact the reproducibility of organoid formation and function (Manduca et al., 2023). Therefore, it is essential to establish uniform methods to ensure consistent and reliable data generation. Additionally, scaling up PDO production poses challenges, especially for aggressive cancers where prompt therapeutic decisions are vital. High-throughput drug screening integration requires cost-effective automation technologies that can maintain patient-specific characteristics (Gjorevski et al., 2016).

Research advancements are aimed at mitigating these limitations. Bioprinting and improved culture media formulations are being developed to enhance PDO viability and accurately replicate the tumor microenvironment (Geyer and Queiroz, 2021). Microfluidic platforms for high-throughput drug screening show promise for efficient analysis of PDOs (Xu et al., 2019b). The role of the tumor microenvironment is pivotal, particularly in influencing the immune response in cancer research. Current PDO models lack continuous blood flow and the diverse cellular components found *in vivo*. This can be addressed by incorporating immune cells, endothelial cells, and stromal components into the organoid culture to better simulate physiological conditions (Choo et al., 2021).

The ongoing research is improving the fidelity of PDOs used in cancer research by addressing their limitations in simulating the TME. Bioprinting technologies show promise in reconstructing the TME with greater accuracy, leading to more representative organoid models (Mi et al., 2023). Researchers are developing advanced culture media that better support PDO viability by mimicking interactions with stromal cells and key signaling molecules (Manduca et al., 2023). Additionally, microfluidic platforms are being explored for their potential in high-throughput drug screening, enabling efficient and cost-effective analysis of PDOs (Geyer and Queiroz, 2021; Yoon et al., 2024).

The effectiveness of cancer immunotherapies is closely linked to the TME’s role in modulating the immune response. However, current PDO models lack continuous blood flow, which is essential for immune cell recruitment (Wang et al., 2022b; Peng et al., 2022). This limitation can be addressed by co-culturing PDOs with immune cells to reflect dynamic tumor–immune interactions (Peng et al., 2022). Nevertheless, traditional PDOs often exclude non-tumor cellular components of the TME, which play a crucial role in shaping immune responses through mechanisms like hypoxia and the presence of immunosuppressive cells (Wang et al., 2021). To capture the complexity of the TME, research has focused on integrating immune and endothelial cells into organoid cultures (Wang et al., 2022b; Peng et al., 2022). Furthermore, investigations are underway for tumor microenvironment-responsive nanomedicines to regulate the TME and enhance immunotherapy outcomes (Wang et al., 2021; Wang et al., 2022b). These advancements are crucial in creating physiologically relevant *in vitro* models that can more accurately mimic the intricate interactions between tumors and the immune system in cancer research. As technology progresses, these improvements in PDO models have the potential to revolutionize personalized cancer therapy and drug development, bringing us closer to clinical applications that replicate the true complexity of human cancers.

## 8 Overcoming barriers to implementation

Biotechnology innovators are using PDOs to advance cancer therapy and research. However, there are challenges in integrating PDOs into drug development. For instance, researchers are developing new biomaterials to better recreate the tumor microenvironment, improving the accuracy and functionality of PDOs (Joyce et al., 2017; Abdul Samat and Yahaya, 2022). Genetic sequencing and editing techniques are also being employed to maintain the genetic similarity between PDOs and the original tumors, address intratumor heterogeneity, and make organoid technologies more relevant (Landon Brace et al., 2021). Improvements in scaffold design contribute to the structural complexity of PDOs, resulting in more reliable outcomes in drug testing (Unnikrishnan et al., 2021). Establishing interdisciplinary consortia with scientists, clinicians, and pharmaceutical companies is crucial for standardizing data sharing and validating organoid models. These networks also create open-source databases, which refine organoid methodologies and boost research productivity (Winkelmaier and Parvin, 2021; Castro Fernandez, 2023). Looking ahead, technological advancements will reshape the PDO landscape. Automated systems are being developed for high-throughput organoid analysis, speeding up drug discovery and pharmacogenomic medicine (Lim et al., 2023; Wijler et al., 2023). AI-driven tools will streamline the assimilation of complex organoid data, optimizing the identification and formulation of targeted treatments (Zhou et al., 2021; Lim et al., 2023). Furthermore, integrating microfluidics enables real-time assessment of drug interactions within PDO cultures, enhancing the predictive power of these models (Deliorman et al., 2023). Overall, these innovations lay the foundation for incorporating PDOs into personalized cancer treatment, overcoming current obstacles and paving the way for future breakthroughs.

## 9 Case studies and clinical trials

PDOs are becoming increasingly important in oncology research, particularly in clinical trials. They have the ability to customize cancer treatment and accelerate drug development. Various studies, such as trial NCT04212545, are validating their role by assessing the use of PDOs to determine the most effective chemotherapy regimens for metastatic gastrointestinal cancers. This has the potential to improve treatment specificity and patient outcomes (Ooft et al., 2019). Another trial, NCT03827850, is utilizing PDOs to identify optimal treatments for patients with advanced solid tumors, demonstrating the shift toward personalized medicine through the utilization of individual tumor biology (Pacini et al., 2021). The potential of PDOs is highlighted by early successes, such as the work of Vlachogiannis et al., which showcased their usefulness in predicting patient-specific responses to anticancer drugs in metastatic gastrointestinal cancer, facilitating customized therapies (Vlachogiannis et al., 2018). However, the widespread integration of PDOs into clinical practice faces challenges. These include the need for scalable production and the development of automated systems for PDO management as well as the establishment of robust regulatory frameworks to ensure a smooth transition into clinical workflows (van Gool et al., 2017). Multi-stakeholder collaborations are crucial in this regard, with initiatives like the OncoTrack project leading the way in standardizing PDO production, ensuring quality control, and validating their use in pharmaceutical medicine (Dantes et al., 2023). Furthermore, cutting-edge technologies such as single-cell analysis and organ-on-a-chip models are advancing PDO technology beyond clinical trials. These technologies provide more detailed insights into tumor heterogeneity and interactions within the microenvironment. This could lead to highly accurate and representative PDO models for personalized cancer treatment and drug development.

In summary, PDOs hold significant promise for the future of personalized cancer care. Ongoing trials and collaborative efforts are establishing a strong foundation for their integration into clinical practice. Continued research and innovation are poised to revolutionize oncology, offering individualized treatment approaches that enhance patient outcomes and redefine the landscape of cancer treatment.

## 10 The road ahead: future perspectives

PDOs are ushering in a new era in oncology, with the potential to revolutionize the development, personalization, and evaluation of cancer therapies (Vlachogiannis et al., 2018). What sets PDOs apart is their ability to study the intricacies of individual tumors in a controlled setting, bridging the gap between traditional cell culture and *in vivo* studies. This high-fidelity nature enhances our understanding of tumor biology, including mechanisms of drug resistance and metastasis. In the field of drug development, PDOs enable efficient screening of therapeutics, potentially reducing the industry’s high attrition rates and expediting the transition from laboratory to clinical use (Zhou et al., 2021; Lim et al., 2023). Technological advancements have expanded the utility of PDOs. Techniques such as CRISPR-Cas9 gene editing provide precise models of genetic mutations, while single-cell RNA sequencing combined with machine learning algorithms show promise in unraveling tumor heterogeneity and predicting patient-specific drug responses (Vlachogiannis et al., 2018; Liu et al., 2021; Chen et al., 2023). Future research will focus on improving co-culture techniques that incorporate immune and stromal components, which are crucial for immuno-oncology studies (Vlachogiannis et al., 2018). Standardizing protocols for PDO generation and maintenance will likely enhance reproducibility and broaden their application in research settings (Lim et al., 2023). The envisioned use of PDOs in clinical practice goes beyond drug development and extends to guiding clinical decision-making. By determining the most effective treatment regimens for individual patients, PDOs could reduce the current trial-and-error approach in oncology, thus improving patient care (Dantes et al., 2023). The predictive capabilities of PDOs have the potential to significantly enhance patient quality of life and survival rates by avoiding ineffective and harmful treatments. Looking forward, the integration of PDOs into diagnostic processes may provide insights into disease progression and recurrence, paving the way for proactive treatment strategies (Aberle et al., 2018). However, realizing this vision requires overcoming challenges such as ethical considerations, scaling up processes, and ensuring equitable access to these innovative tools. Despite these obstacles, the transformative potential of PDOs for patient care remains unparalleled.

## 11 Conclusion

PDOs have the potential to greatly advance personalized cancer treatments. These three-dimensional models are directly cultivated from a patient’s cancer cells, providing a more accurate representation of the patient’s unique tumor biology compared to traditional 2D cultures or animal models. This personalized approach allows for targeted evaluation of drug efficacy and the study of resistance mechanisms, tailoring treatment to the patient’s genetic profile and enhancing oncological precision. Despite the excitement surrounding PDOs for their role in cancer drug discovery and the potential for more customized therapies, several challenges remain. Issues such as cost, scalability, and accurately replicating the complexity of tumor–stroma interactions within the organoids must be addressed. While the use of PDOs in clinical trials is still in its early stages, the increasing sophistication of these models, which increasingly resemble *in vivo* conditions, highlights their growing importance. With ongoing investment and research, PDOs are poised to revolutionize cancer care and become a crucial component of the oncology arsenal, ultimately improving patient outcomes.
